# A comparison of sunlight exposure in men with prostate cancer and basal cell carcinoma

**DOI:** 10.1038/sj.bjc.6603576

**Published:** 2007-01-30

**Authors:** N J Rukin, M P Zeegers, S Ramachandran, C J Luscombe, S Liu, M Saxby, J Lear, R C Strange

**Affiliations:** 1 Human Disease and Genomics Research Group, Institute of Science and Technology in Medicine, Keele University Medical School, University Hospital of North Staffordshire, Staffordshire, ST4 7PX, UK; 2 Department of Urology, University Hospital of North Staffordshire, Staffordshire, ST4 7PX, UK; 3 Unit of Genetic Epidemiology, Department of Public Health and Epidemiology, University of Birmingham, Edgbaston, Birmingham, B15 2TT, UK; 4 Department of General Practice, Catholic University of Leuven, Belgium; 5 Department of Biochemistry, Good Hope Hospital, Birmingham, B75 7RR, UK; 6 Dermatology Centre, Hope Hospital, Salford, Manchester, M6 8HD, UK

**Keywords:** prostate cancer, basal cell carcinoma, benign prostatic hypertrophy, ultraviolet radiation, exposure patterns, disease susceptibility

## Abstract

Ultraviolet radiation exposure increases basal cell carcinoma (BCC) risk, but may be protective against prostate cancer. We attempted to identify exposure patterns that confer reduced prostate cancer risk without increasing that of BCC. We used a questionnaire to assess exposure in 528 prostate cancer patients and 442 men with basal cell carcinoma, using 365 benign prostatic hypertrophy patients as controls. Skin type 1 (odds ratio (OR)=0.47, 95% CI=0.26–0.86), childhood sunburning (OR=0.38, 95% CI=0.26–0.57), occasional/frequent sunbathing (OR=0.21, 95% CI=0.14–0.31), lifetime weekday (OR=0.85, 95% CI=0.80–0.91) and weekend exposure (OR=0.79, 95% CI=0.73–0.86) were associated with reduced prostate cancer risk. Skin type 1 (OR=4.00, 95% CI=2.16–7.41), childhood sunburning (OR=1.91, 95% CI=1.36–2.68), regular foreign holidays (OR=6.91, 95% CI=5.00-9.55) and weekend (OR=1.17, 95% CI=1.08–1.27) but not weekday exposure were linked with increased BCC risk. Combinations of one or two parameters were associated with a progressive decrease in the ORs for prostate cancer risk (OR=0.54–0.25) with correspondingly increased BCC risk (OR=1.60–2.54). Our data do not define exposure patterns that reduce prostate cancer risk without increasing BCC risk.

Ultraviolet radiation (UVR) has deleterious and beneficial effects, and humans have developed phenotypes that mediate these effects (Wong and Rees, 2005). Thus, UVR is a key factor in the pathogenesis of skin cancers such as basal cell carcinoma (BCC) (Karagas and Greenberg, 1995). However, the pattern and intensity of exposure needed for BCC development is uncertain. For example, although evidence linking total sun exposure to BCC risk is weak, childhood sunburning and infrequent, possibly intense bursts of UVR rather than a similar continuous dose appear important (Karagas and Greenberg, 1995; Kricker *et al*, 1995; Corona *et al*, 2001). Thus, intermittency characterised by high weekend relative to weekday exposure is an important concept in BCC development (Kricker *et al*, 1995).

Ultraviolet radiation exerts beneficial effects such as the initiation in skin of the 1,25-dihydroxy vitamin D synthetic pathway. The recognition that 1,25-dihydroxy vitamin D exerts a key role in numerous biochemical pathways (Holick, 2005), and that hypovitaminosis is common worldwide (Calvo *et al*, 2005) has contributed to the idea that low levels of exposure confer increased risk of various diseases including some cancers (Grant, 2002). For example, we found regular holidays abroad and sunbathing were inversely associated with prostate cancer risk (Luscombe *et al*, 2001; Bodiwala *et al*, 2003). Independent studies have reported inverse associations between exposure and serum vitamin D levels and prostate cancer risk and mortality (Hanchette and Schwartz, 1992; John *et al*, 2004; Tuohimaa *et al*, 2004; John *et al*, 2005; Schwartz, 2005; Colli and Colli, 2006).

If chronically low levels of exposure are linked with increased risk of diseases such as prostate cancer, it is important to determine if there is an exposure pattern that allows adequate vitamin D synthesis, without risking skin cancer. We describe a questionnaire-based study that compares exposure in men with prostate cancer, BCC and benign prostatic hypertrophy (BPH). These diseases were selected because they are common and have been studied in the context of the UVR/disease risk hypothesis (Armstrong and Kricker, 2001; Luscombe *et al*, 2001). Our aims were to firstly, compare exposure patterns in these groups, secondly, determine which combinations of exposure parameters were most associated with prostate cancer and BCC risk and thirdly, determine if exposure patterns could be identified that were associated with reduced risk of prostate cancer, without increasing the risk of BCC.

## MATERIALS AND METHODS

### Patients

The study group comprised prevalent cases of prostate cancer (528), BPH (365) and BCC (442) in the northwest of England and attending the University Hospital of North Staffordshire or Dermatology Centre, Hope Hospital, Manchester. These hospitals are approximately 40 miles apart. All subjects were male, unrelated, British Northern European Caucasians recruited with Ethics Committee approval and written, informed consent. Prostate cancer and BPH patients were resident in North Staffordshire, an area with a stable and homogeneous population, and were recruited in urology clinics in the University Hospital of North Staffordshire (Luscombe *et al*, 2001; Bodiwala *et al*, 2003). We recruited about 70% of suitable men. Five men (four BPH, one cancer) declined participation. Men came via prostatic-specific antigen (PSA) screening or were referred for lower urinary tract symptoms. Diagnosis of prostate cancer was performed histologically after trans-rectal ultrasound biopsy (TRUS biopsy) or trans-urethral resection of prostate (TURP). Benign prostatic hypertrophy men had PSA values in the reference range, non-malignant digital rectal examination and non-malignant histology. Characteristics of cancer cases, typical for a UK hospital, were; 43.7% had advanced stage disease and 34.0% a Gleason sum 8–10. Sixteen prostate cancer and seven BPH patients had a BCC and two BPH patients malignant melanoma. They were excluded from the analysis.

Four hundred and forty-two males with one or more histologically proven BCC were recruited at the first presentation or during follow-up at the Dermatology Centre, Manchester from the approximately 1200 patients who attended the Centre during 2000–2004 (Lovatt *et al*, 2005). Patients not recruited included those who were randomly missed in busy clinics, those who refused to participate and those with other serious pathology, including basal cell naevus syndrome or xeroderma pigmentosum. Two BCC cases with prostate cancer were excluded from analysis.

### UVR exposure

Cases completed, at recruitment, an identical questionnaire that recorded the parameters of acute and chronic UVR exposures (Luscombe *et al*, 2001; Bodiwala *et al*, 2003). The questionnaire records (i) skin type: type 1, always burn/never tan; type 2, usually burn/tan with difficulty; type 3, sometimes mild burn/average tanning ability; type 4, rarely burn/easily tan (Fitzpatrick, 1988); (ii) cumulative exposure per day determined by adding the hours exposure on each weekday and weekend in the following age categories: early adulthood (ages 20–39.9), mid adulthood (ages 40–59.9) and late adulthood (aged over 60). These data were combined to give the mean hours day^−1^ exposure between 20 years of age to age at diagnosis; (iii) childhood sun burning, defined as erythema for over 48 h or blistering (yes/no); (iv) regular foreign holidays were defined as one or more foreign holidays in a sunny climate per year for 10 years (yes/no); (v) weekday and weekend exposure in mean hours day^−1^ were derived from exposures in the three age categories. Few cases reported the use of sunscreens and no case regularly used a sun bed. Questionnaires were self-administrated to avoid interviewer bias.

### Statistical analysis

Benign prostatic hypertrophy cases were used as controls as they are a good representation of the older male population (Berry *et al*, 1984). Logistic regression analysis was used to compare UVR exposure parameters in the groups. Interactions between age, skin type and variables of exposure were identified and these confounders were adjusted for in logistical regression models. To predict which exposure parameters were most significantly associated with risk, the statistical significance of logistic regression models was assessed. The difference in log likelihood between the sub model and full model is approximately distributed as *χ*^2^, with degrees of freedom (df) equal to the difference in the number of covariates between the sub model and the full model. Akaike's information criteria (AIC=*χ*^2^−2df) was used to derive suitable multivariate statistical models for each disease (Akaike, 1987). The sub model with the lowest AIC reflects the best balance between goodness of fit and parsimony. Statistical analyses were performed using Stata version 8 (Stata Corporation, College Station, TX, USA).

## RESULTS

### Skin type, exposure parameters and prostate cancer and BCC risk

Table 1 shows details of age, skin type and exposure parameters in the three patient groups. The mean age of BPH cases was lesser than that of prostate cancer and BCC cases. Accordingly, age-adjusted odds ratios (OR) were obtained by including age as a continuous variable in all models. Table 2 shows the results of logistic regression analyses used to compare the proportions of men with skin types 1–4 and exposure parameters in BPH, and prostate cancer or BCC cases. Skin type was associated with disease susceptibility; relative to type 4 (reference category), in prostate cancer cases, type 1 was associated with significantly reduced disease risk (OR=0.47, 95% CI=0.26–0.86) whereas in BCC cases, types 1, 2 and 3 were associated with increased risk (OR=2.40–4.00). Logistic regression models were therefore adjusted for skin type (factorised as types 1, 2, 3 and 4) in addition to age.

Parameters of exposure to UVR were also associated with risk. Table 1 shows that 101/528 (19.1%) prostate cancer and 325/442 (73.5%) BCC cases reported regular foreign holidays. This activity was associated with reduced prostate cancer (OR=0.58, 95% CI=0.42–0.80) but increased BCC (OR=6.91, 95% CI=5.00–9.55) risk (Table 2). Similarly, prostate cancer risk was reduced (OR=0.38, 95% CI=0.26–0.57) in men who reported sunburn in childhood (48/528 cases, 9.1%), whereas BCC risk was increased (OR=1.91, 95% CI=1.36–2.68) in those who were sunburnt (162/442 cases, 36.7%).

Increased daily exposure (hours/day) was also associated with reduced prostate cancer (OR=0.78, 95% CI=0.72–0.85) and increased BCC (OR=1.08, 95% CI=1.00–1.17) risk. Because BCC risk has been associated with weekend rather than weekday exposure, we determined the association of prostate cancer and BCC risk with these exposures separately. Both weekend and weekday exposures were similarly associated with reduced prostate cancer risk, although BCC risk was significantly associated with weekend (OR=1.17, 95% CI=1.08–1.27) but not weekday (OR=1.00, 95% CI=0.94–1.06) exposure (Table 2).

### Selection of the predictive factors for prostate cancer and BCC risk

The AIC was used to identify the most significant covariates for the two cancers from predictive models comprising age at diagnosis, skin type (type 4 *vs* types 1–3), regular foreign holidays, childhood sunburn, average sunbathing score (never *vs* rarely/occasional/frequent) and average weekday and average weekend exposures. Akaike's information criteria-guided model selection resulted in skin type being dropped from the predictive multivariate model for prostate cancer (OR=0.76, 95% CI=0.53–1.13, *P*=0.190) and average sunbathing score (OR=0.74, 95% CI=0.48–1.13, *P*=0.167) being dropped from the model for BCC. Odds ratios derived for multivariate analysis for prostate cancer and BCC are shown in Table 3. We did not identify colinearity between any combinations of the studied variables in these models.

To determine if the effects of weekday and weekend exposures are different in prostate cancer and BCC, we removed average weekday exposure from the AIC-derived models for prostate cancer and BCC. This resulted in a significant change in the log likelihood value (*P*=0.0280) of the regression model for prostate cancer while for BCC, we observed a change in the log likelihood value that approached but did not achieve significance (*P*=0.0782). These findings suggest that the effects of weekday and weekend exposure are different in prostate cancer and possibly different in BCC.

### Association of combinations of variables on prostate cancer and BCC risk

**Figure 1:**
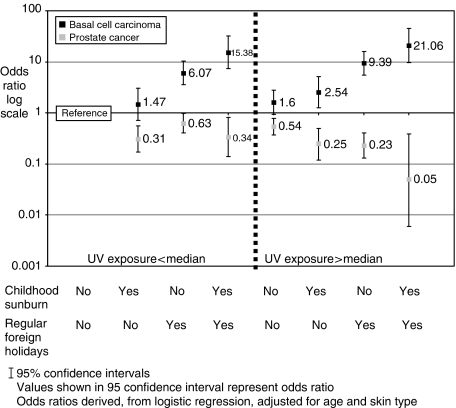
Log scale odds ratios for predictive models split by median weekend UVR exposure, childhood sunburning and regular foreign holidays in prostate cancer and male BCC patients

We further investigated the association of childhood sunburn (yes/no), foreign holidays (yes/no) and exposure at weekends (<6/>6 h weekend^−1^; median value in BPH cases 6 h) with risk by comparing the effect of combinations of these variables on prostate cancer or BCC risk. As UVR exposure interacts with childhood sunburn and regular foreign holidays, the ORs for combinations of these factors cannot be predicted from the individual ORs. Figure 1 shows the association of combinations of childhood sunburning and foreign holidays in men stratified by the median value of weekend exposure. Combinations of one or two of the parameters were associated with a progressive decrease in OR values for prostate cancer risk. Correspondingly, such combinations were associated with increased BCC risk. Regular foreign holidays appeared to be particularly associated with BCC risk (Figure 1).

## DISCUSSION

The observation that hypovitaminosis D is widespread together with the suggestion that this phenotype is associated with a significantly increased risk of various diseases has potential public health implications (Calvo *et al*, 2005; Holick, 2005; Reichrath, 2006). Indeed, it has been suggested controversially that attitudes to sun exposure should change (Grant, 2002; Lucas *et al*, 2006). Clearly, the definition of a pattern/level of exposure that allowed adequate synthesis of vitamin D without increasing skin cancer risk would be useful. Presumably, this putative level will be determined by the intensity and duration of exposure and host factors such as skin pigmentation (Bodiwala *et al*, 2003); deeply pigmented skin has a sun protective factor of about 10 compared with pale skin (Cripps, 1981). Accordingly, we have defined patterns of exposure associated with reduced prostate cancer and increased BCC risk and considered these to determine whether distinct, disease-specific patterns of exposure can be defined.

Both case diseases are increasingly common and present substantial problems to health care agencies. Thus, BCC incidence is increasing with an estimated one if three lifetime risk for Caucasian Americans born in the 1990s. While exposure to UVR is recognised as the key causative factor, the relationship is complex; for example, the frequency of lesions on the face does not correlate with site-specific exposure (Heckmann *et al*, 2002). Prostate cancer appears to result equally from genetic and environmental factors (Lichtenstein *et al*, 2000). It is the cause of 13% of male cancer deaths with a lifetime risk of 1 in 13 (Office for National Statistics, 2005). We used BPH as the comparison group as it is common (prevalence in men aged 50 years about 50%) and part of normal ageing. BPH is not believed to be associated with increased prostate cancer risk or vitamin D status (Young *et al*, 2000). Importantly, these men had been investigated for BCC as well as prostate cancer.

Assessing the intensity/duration of lifetime exposure using questionnaire-derived data is problematic. Thus, both the reliability (reproducibility) of data describing past exposure in a predominantly elderly subject group as well as the possibility of recall bias in cases and controls may be questioned. Some studies have found good agreement between repeat responses and no evidence of bias between multiple sclerosis and skin cancer cases and their respective controls (Rosso *et al*, 2002; van der Mei *et al*, 2006). However, some parameters such as number of lifetime sunburns (but not childhood sunburning) and intermittency generally demonstrated weaker agreement (English *et al*, 1998; Rosso *et al*, 2002; Relova *et al*, 2005; van der Mei *et al*, 2006). Other approaches such as use of an index based on the difference in pigmentation in the less exposed underarm skin (constitutive pigmentation) and widely exposed forehead (facultative pigmentation) have been criticised (Oh *et al*, 2004). Light sensitive meters provide only acute data and may not reflect the fact that body sites differ in ability to synthesise vitamin D, with the trunk being more effective than the head/neck.

We collected data on aspects of exposure linked with BCC and prostate cancer risk (Karagas and Greenberg, 1995; Luscombe *et al*, 2001; Bodiwala *et al*, 2003). While the mechanism for associations between UVR and prostate cancer risk is unclear it may be related to UVR-mediated synthesis of vitamin D in skin. Thus, we believe that the exposure parameters are surrogates for chronic vitamin D status. Importantly, the relationship between duration and intensity of exposure and vitamin D synthesis is complex and latitude dependent. Indeed, while regular, short periods of exposure appear to allow adequate synthesis, worldwide hypovitaminosis has been reported (Calvo *et al*, 2005). Even substantial periods outdoors may not allow adequate vitamin D synthesis. Thus, while exposures of only 10 min at midday may be adequate in sunny climates throughout the year, no synthesis occurs during winter in northern Europe (Holick, 2005). Indeed, exposure patterns such as sunbathing may be more effective than longer periods outside with only the face exposed.

Cumulative exposure/year assesses occupational and recreational activity. Sunbathing allows exposure of a large skin area, and holidays in hot climates can allow short-term continuous, intense exposure. Individually these parameters may not fully describe low or high exposures; limited time in the sun might allow adequate vitamin D synthesis if sufficient area is regularly exposed around midday (Luscombe *et al*, 2001). The parameters we studied were weakly correlated indicating each assessed different aspects of exposure and all contributed to an overall index (Bodiwala *et al*, 2003; Lovatt *et al*, 2005). BCC risk is related to sunburning in childhood and intermittent exposure; weekend (recreational) rather than weekday (occupational) exposure is important in determining risk (Karagas and Greenberg, 1995; Kricker *et al*, 1995; Corona *et al*, 2001; Wong *et al*, 2003). These associations were observed in our data. The data in Figure 1 also demonstrated that holidays in hot climates each year for at least 10 years which might be considered as an intense form of intermittent exposure were strongly associated with BCC risk. Our previous studies showed that several parameters of exposure were significantly associated with reduced prostate cancer risk (Luscombe *et al*, 2001; Bodiwala *et al*, 2003). Mechanistically this may indicate that any pattern of exposure is protective as long as it allows adequate vitamin D synthesis. Interestingly, weekend exposure, on the basis of the values of the ORs, appeared to offer more effective protection against prostate cancer. Our analysis indicated that parameters of exposure considered individually and in combination were equally associated with decreased prostate cancer or increased BCC risk. Indeed, inspection of Figure 1 shows that none of the parameters studied allows exposure that is without consequences in terms of BCC risk. Importantly, the ORs presented are corrected for skin type and it is possible that the impact of UVR on risk may be markedly different in men with sun sensitive types 1 and 2 compared with types 3 and 4.

In conclusion we have shown that similar exposure patterns determine reduced prostate cancer and increased BCC risk. The data presented do not allow us to define levels of exposure that reduce prostate cancer risk without increasing BCC risk.

## Figures and Tables

**Table 1:** Exposure parameters in prostate cancer, male basal cell carcinoma patients and benign prostatic hypertrophy patients

	**Prostate cancer (n=*528*)**	**Basal cell carcinoma (n=*442*)**	**Benign prostatic hypertrophy (n=*365*)**
Age (years)	70.2±7.72 (range 35–91)	68.8±10.33 (range 32–90)	66.8±8.10 (range 46–86)
			
*Skin type* a			
1 (never tans)	29 (5.5%)	62 (14.0%)	32 (8.8%)
2	152 (28.8%)	158 (35.8%)	109 (29.9%)
3	234 (44.3%)	192 (43.4%)	165 (45.2%)
4 (readily tans)	113 (21.4%)	30 (6.8%)	59 (16.2%)
			
*Foreign holiday* b			
No/Yes	427/101	117/325	251/114
			
*Childhood sunburn* c			
No/Yes	480/48	280/162	278/87
			
*Sunbathing*			
Never	201 (38.1%)	80 (18.1%)	61 (16.7%)
Rarely	214 (40.5%)	172 (38.9%)	140 (38.4%)
Occasional/frequent	113 (21.4%)	190 (43.0%)	164 (44.9%)
			
Average daily sun exposure^d^	3.63±1.66	4.63±2.02	4.36±1.69
Average weekday exposure^d^	3.35±2.00	4.08±2.40	4.06±2.20
Average weekend exposure^d^	3.91±1.71	5.19±2.07	4.66±1.69

± Represents s.d.

a Numbers of patients.

b Defined as one or more foreign holidays in a sunny climate per year for 10 years.

c Childhood sunburn represents one or more episodes of skin blistering or sunburn lasting more than 48 h.

d Hours per day.

**Table 2:** Skin type and ultraviolet radiation exposure parameters for prostate cancer and male BCC patients

	**Prostate cancer OR (95% CI)**	***P*-value**	**Basal cell carcinoma OR (95% CI)**	***P*-value**
*Skin type*				
1(never tans)	0.47 (0.26–0.86)	0.031	4.00 (2.16–7.41)	<0.001
2	0.73 (0.49–1.09)	0.120	3.13 (1.88–5.21)	<0.001
3	0.74 (0.51–1.07)	0.114	2.40 (1.47–3.92)	<0.001
4 (readily tans)	Reference		Reference	
*P*-trend		0.024		<0.001
				
*Foreign Holiday*^a^^,^^b^				
No	Reference		Reference	
Yes	0.58 (0.42–0.80)	0.001	6.91 (5.00–9.55)	<0.001
				
*Childhood sunburn*^a^^,^^c^				
No	Reference		Reference	
Yes	0.38 (0.26–0.57)	<0.001	1.91 (1.36–2.68)	<0.001
				
*Sunbathing*^a^				
Never	Reference		Reference	
Rarely	0.46 (0.32–0.67)	<0.001	1.13 (0.75–1.71)	0.561
Occasional/frequent	0.21 (0.14–0.31)	<0.001	1.27 (0.84–1.94)	0.260
Average daily sun exposure^a^^,^^d^	0.78 (0.72–0.85)	<0.001	1.08 (1.00–1.17)	0.039
Average weekday exposure^a^^,^^d^	0.85 (0.80–0.91)	<0.001	1.00 (0.94–1.06)	0.977
Average weekend exposure^a^^,^^d^	0.79 (0.73–0.86)	<0.001	1.17 (1.08–1.27)	<0.001

Abbreviations: CI=confidence interval; OR=odds ratio.

a Adjusted for age and skin type.

b Defined as one or more foreign holidays in a sunny climate per year for 10 years.

c Childhood sunburn represents one or more episodes of skin blistering or sunburn lasting more than 48 h.

d Hours per day.

**Table 3:** Multivariate analysis using predictive models to assess risk in prostate cancer and basal cell carcinoma

	**Prostate cancer OR (95% CI)**	***P*-value**	**Basal cell carcinoma OR (95% CI)**	***P*-value**
Age at diagnosis	1.03 (1.01–1.05)	0.002	1.05 (1.03–1.07)	<0.001
Skin type	—		2.58 (1.52–4.39)	<0.001
Foreign holidays	0.64 (0.46–0.90)	0.010	6.38 (4.61–8.83)	<0.001
Childhood sunburn	0.35 (0.23–0.52)	<0.001	2.08 (1.44–3.01)	<0.001
Average sunbathing score	0.39 (0.27–0.55)	<0.001	—	
Average weekend exposure	0.85 (0.77–0.95)	0.003	1.22 (1.09–1.36)	<0.001
Average weekday exposure	0.91 (0.84–0.99)	0.028	0.93 (0.85–1.01)	0.086
Akaike information criteria (AIC)^a^	1082.37		914.67	
score				

Abbreviations: CI=confidence interval; OR=odds ratio.

a Multivariant analysis derived from Akaike's information criteria using a model comprising age at diagnosis, skin type (skin type 4 *vs* skin type 1–3), regular foreign holidays, childhood sunburn, average sunbathing score (never *vs* rarely/occasional/frequent) and average weekday and weekend exposures.
